# Pregnancy among people with TB disease

**DOI:** 10.5588/ijtldopen.25.0381

**Published:** 2026-04-13

**Authors:** K. Wendorf, C. Dollinger, P. Barry, A. Readhead

**Affiliations:** California Department of Public Health, Tuberculosis Control Branch, Richmond, CA, USA.

**Keywords:** tuberculosis, screening, prevention, TB infection

## Abstract

**BACKGROUND:**

Globally, TB disproportionately impacts people of childbearing age. However, the impact of pregnancy among people with TB in California is unknown.

**METHODS:**

Women aged 15–49 years with TB in California during 1993–2019 were matched to California birth records to identify those whose pregnancies or postpartum periods overlapped with TB treatment. Demographic and clinical variables were compared by pregnancy status, which included ‘peri-pregnant’ (all pregnant categories), ‘pre-pregnant’ (treatment started before and continued during pregnancy), ‘pregnant’ (treatment started during pregnancy), and ‘postpartum’ (treatment started within 3 months of a live birth).

**RESULTS:**

Pregnancy and TB co-occurred among 7% of 15,092 individuals. Peri-pregnant people were more likely to be under 30 years old (*P* < 0.001), have recently immigrated (*P* < 0.001), and have pulmonary TB (*P* < 0.001) than nonpregnant people. Pregnant and postpartum people were 2.3 times more likely to have a TB diagnosis lacking microbiological confirmation than pre-pregnant people (95% confidence interval [1.7–3.2]). Pyrazinamide was used for >90% of pre-pregnant and postpartum groups, but 41% (*P* < 0.001) for people started on TB treatment while pregnant.

**CONCLUSION:**

Pregnancy occurs commonly among people of childbearing age with TB in California. Current TB screening practices among pregnant patients might prevent or detect early TB disease.

Pregnant people with TB battle a disease that threatens both their lives and their foetuses’ lives. They might be socially isolated to limit risk of transmission and face this challenge alone. Globally, women disproportionately develop TB disease between the ages of 15 and 34^1^; most reside in the WHO African and Southeast Asian regions.^2^ Prevalence of active TB among pregnant patients has been estimated to be as high as 11% among HIV-positive people in high-incidence TB countries, with significantly lower rates in low-burden countries like the USA (0.06%–0.25%).^3^ There is disagreement regarding whether pregnancy itself increases susceptibility to TB infection or risk of disease progression.^3–5^ It appears clear that there is an increased risk of progression from latent TB infection (LTBI) to active TB disease during the postpartum period.^5–9^ TB can be harder to diagnose during pregnancy because TB symptoms can be mistaken for typical pregnancy symptoms.^3,7^ Non-specific symptoms can result in diagnostic delays during pregnancy and the early postpartum period.^10,11^. When identified and treated appropriately, pregnant people can have high TB treatment success rates.^12^

In the USA, pregnant people are recommended to be screened for TB risk factors during early pregnancy. Those with TB risk are recommended to undergo TB testing and a chest X-ray (CXR) to evaluate for pulmonary TB disease if the TB test is positive.^13^ The California and US surveillance systems for TB have only recently started to collect pregnancy status, so the co-occurrence of TB and pregnancy in California is not well studied. Our goal was to understand the incidence of pregnancy among TB patients in California over the last three decades and the incidence of TB diagnoses occurring during the early postpartum period. Secondary goals were to describe the demographics, clinical characteristics, and TB outcomes of pregnant versus nonpregnant people with TB to inform TB prevention practices.

## METHODS

Data on persons with TB are reported to the California Department of Public Health by local health departments (LHDs). We included patients aged 15–49 reported as women and diagnosed with TB during 1993–2019. TB diagnosis date was defined as the earliest of the following: treatment start, collection of first positive culture, collection of first positive nucleic acid amplification test, or date reported by LHDs. We matched TB patients to California birth certificate data on mother’s first name, last name (mother’s birth surname and baby’s surname on birth certificate), date of birth, race, and zip code, using Match*Pro v2.0.6 (National Cancer Institute). We required an exact match on at least one of first name, last name, or date of birth. We further restricted to those taking active TB treatment at any point during pregnancy or within 3 months of delivery; included were TB patients diagnosed on or after 3/26/1993 (90 days after the first available birth data) and who completed treatment by 3/27/2020 (40 weeks before the last available birth data). We matched individuals to an outbreak dataset to identify involvement in an outbreak. Data on age and race/ethnicity of California mothers from 1995 to 2020 were downloaded from CDC WONDER; data were not available for 1993–1994.^14^

People were classified as ‘peri-pregnant’ if they were on TB treatment at any point during pregnancy or started treatment within 3 months of a live birth. People within the ‘peri-pregnant’ category were further subdivided into three categories: pre-pregnant, pregnant, and postpartum to evaluate the potential impact of the timing of pregnancy related to TB treatment. ‘Pre-pregnant’ people started TB treatment prior to conception date (40 weeks prior to delivery date) and continued TB treatment during pregnancy (TB treatment end date was after conception date). ‘Pregnant’ people started TB treatment during pregnancy. ‘Postpartum’ people started TB treatment within 3 months of birth (Figure). The remaining people were classified as not pregnant.

**Figure.**
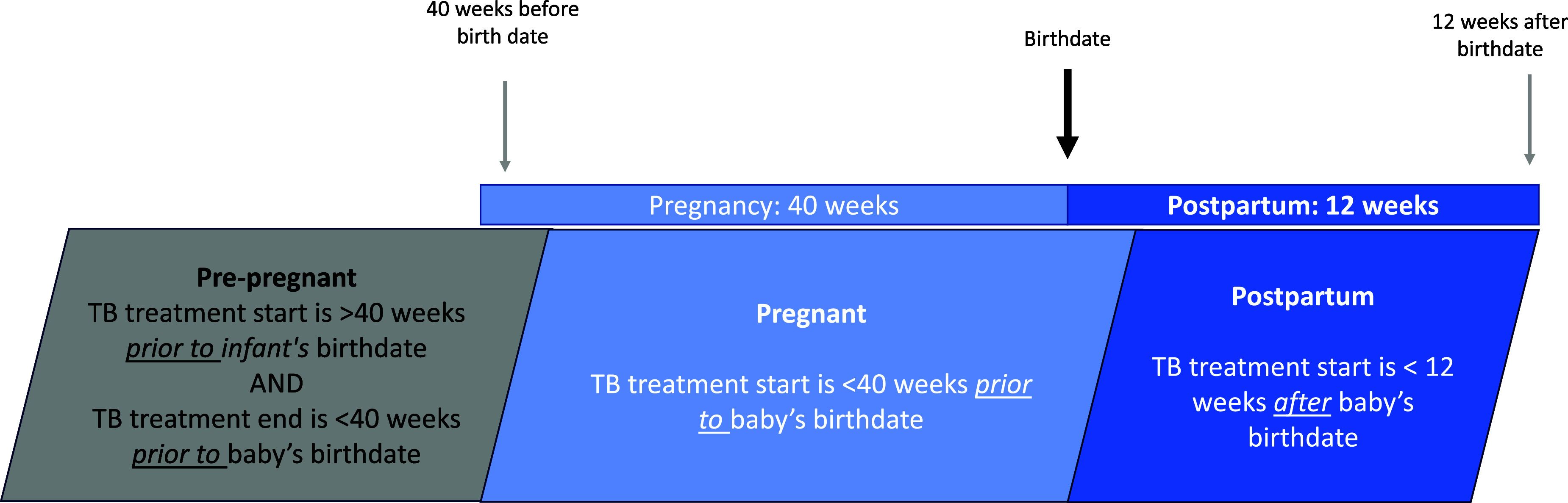
Peri-pregnant groups according to baby’s birthdate and mother’s TB treatment start and end dates.

LHDs were classified into regions. Case verification was dichotomised into microbiologically confirmed (culture positive, AFB smear positive, and/or nucleic acid amplification test positive on any specimen) and not microbiologically confirmed. Patient CXR results were dichotomised into ‘consistent with TB’ or ‘not consistent with TB’. Variables indicating cavitation or miliary TB on CXR were restricted to those who had pulmonary disease with an abnormal CXR. Data elements in the California TB registry have evolved, with some variables added in 2010 (interferon-gamma release assay/tuberculin skin test [IGRA/TST] results, contact to a person with infectious TB) and 2014 (outbreak-associated). We used Pearson’s χ² tests, except for cell sizes below 2 where simulated Fisher’s test was used instead. We used logistic regression to calculate unadjusted and adjusted odds ratios for correlations that were of interest and potentially confounded. The analysis was conducted in SAS Enterprise Guide 7.1 and in R Studio 2022.12.0.

### Ethical statement

This analysis was reviewed and determined to be a nonresearch public health activity by the Committee for the Protection of Human Subjects at the California Health and Human Services Agency.

## RESULTS

We identified 14,004 nonpregnant women and 1,088 (7%) peri-pregnant women aged 15–49 years with TB during 1993–2020 in California (Table 1). Peri-pregnant people were younger than nonpregnant people; 73% of peri-pregnant people were 30 years old or younger compared to 43% of nonpregnant people in this age range (*P* < 0.001). Among peri-pregnant people, 53% were Hispanic compared to only 34% of the nonpregnant cohort (*P* < 0.001). Hispanic people were 2.4 times more likely to be peri-pregnant than non-Hispanic White people even after adjusting by age (95% confidence interval [CI] [1.7–3.5]). Most of the cohort was born outside of the USA (78% of nonpregnant and 82% peri-pregnant, Table 1), consistent with overall TB demographics in California. Peri-pregnant people were more likely to have arrived in the USA in the previous 2 years compared to nonpregnant people (27% vs. 17%; *P* < 0.001). A higher percentage of peri-pregnant people lived in the Central Region of California (*P* < 0.001). Immune-compromising conditions were rare overall; HIV and immune-suppression were more common among nonpregnant people (*P* ≤ 0.001 and *P* = 0.036, respectively, Table 2).

**Table 1.** Demographic characteristics by pregnancy status.

Characteristic	Peri-pregnant, N = 1,088^A^	Not pregnant, N = 14,004^A^	*P* value^B^
Age			<0.001
15–18	66 (7.6%)	803 (92%)	
19–25	400 (13%)	2,728 (87%)	
26–30	330 (12%)	2,525 (88%)	
31–35	200 (8.1%)	2,284 (92%)	
36–40	83 (3.9%)	2,072 (96%)	
41–45	9 (0.4%)	2,021 (100%)	
46–49	0 (0%)	1,571 (100%)	
Race and ethnicity^C^			<0.001
American Indian/Native Alaskan	1 (2.9%)	34 (97%)	
Asian	377 (5.3%)	6,732 (95%)	
Black	83 (5.6%)	1,402 (94%)	
Hispanic	580 (11%)	4,785 (89%)	
Multiracial	0 (0%)	17 (100%)	
Native Hawaiian/Pacific Islander	10 (11%)	84 (89%)	
White	37 (3.9%)	911 (96%)	
Nativity^D^			0.003
Non-US born	894 (7.5%)	10,953 (92%)	
US born	194 (6.0%)	3,018 (94%)	
Time in the USA (non-US born)^E^			<0.001
<2 years	291 (11%)	2,435 (89%)	
2–5 years	207 (9.2%)	2,037 (91%)	
Over 5 years	360 (5.7%)	5,967 (94%)	
Region			0.007
Bay Area	273 (6.4%)	3,979 (94%)	
Northern	3 (3.7%)	79 (96%)	
Central	141 (9.3%)	1,374 (91%)	
Border	123 (7.6%)	1,491 (92%)	
Southern	216 (7.1%)	2,812 (93%)	
Los Angeles	332 (7.2%)	4,269 (93%)	

A n (%).

B Fisher’s exact test for count data with simulated *P* value (based on 2,000 replicates); Pearson’s χ² test.

C 39 records excluded because of missing data on race and ethnicity.

D 33 records excluded because of missing data on nativity.

E Limited to those born outside the USA. 575 records excluded because of missing data on time in the USA (non-US born).

**Table 2.** Clinical characteristics by pregnancy status.

Characteristic	Peri-pregnant, N = 1,088^A^	Not pregnant, N = 14,004^A^	*P* value^B^
TST and/or IGRA positive	860 (79%)	10,216 (73%)	<0.001
Sputum smear positive^C^	370 (40%)	4,743 (41%)	0.001
Microbiologically confirmed^D^	838 (77%)	11,154 (80%)	0.027
Chest X-ray consistent with TB^E^	938 (87%)	10,631 (77%)	<0.001
Chest X-ray, evidence of a cavity^F^	194 (23%)	2,471 (26%)	0.074
Chest X-ray, evidence of miliary TB^G^	3 (1.6%)	52 (2.0%)	>0.9
HIV positive^H^	16 (1.5%)	539 (3.9%)	<0.001
TNF antagonist treatment	0 (0%)	36 (0.3%)	0.11
Post organ transplant	1 (<0.1%)	13 (<0.1%)	>0.9
Diabetes	14 (1.3%)	294 (2.1%)	0.068
End-stage renal disease	0 (0%)	49 (0.3%)	0.048
Immunosuppressed^I^	4 (0.4%)	142 (1.0%)	0.036
Missed contact^J^	1 (<0.1%)	15 (0.1%)	>0.9
Site of disease^K^			<0.001
Extra-pulmonary only	212 (19%)	3,937 (28%)	
Pulmonary and other	65 (6.0%)	989 (7.1%)	
Pulmonary only	811 (75%)	9,077 (65%)	
Meningeal TB	4 (0.4%)	59 (0.4%)	>0.9
*Mycobacterium bovis*	6 (0.6%)	111 (0.8%)	0.4
≥90 days between positive IGRA/TST and treatment start^L^	59 (36%)	556 (23%)	<0.001
Initial treatment includes PZA^M^	813 (75%)	13,198 (94%)	<0.001
Died prior to or during treatment	3 (0.3%)	339 (2.4%)	<0.001
Completed treatment^N^	1,016 (93%)	12,782 (92%)	0.2
Lost	24 (2.2%)	209 (1.5%)	0.07
Other reason for incomplete treatment^O^	45 (4.1%)	606 (4.3%)	0.8
Outbreak-associated^P^	7 (0.6%)	1 (<0.1%)	<0.001

TST = tuberculin skin test; IGRA = interferon-gamma release assay; TNF = tumour necrosis factor; PZA = pyrazinamide.

A n (%).

B Pearson’s χ² test; Fisher’s exact test; Fisher’s exact test for count data with simulated *P* value (based on 2,000 replicates).

C Limited to persons with pulmonary disease. 450 records excluded because of missing data on sputum smear status.

D 56 records excluded because of missing data on microbiologically confirmed.

E 212 records excluded because of missing data on chest X-ray abnormal, consistent.

F Limited to persons with pulmonary disease and an abnormal chest X-ray consistent with TB disease. 20 records excluded because of missing data on abnormal chest X-ray, evidence of a cavity.

G Limited to persons reported with an abnormal chest X-ray consistent with TB disease and whose TB disease was reported from 2010 to 2019 because data on abnormal chest X-ray, evidence of miliary TB, were not collected before 2010. 26 records excluded because of missing data on abnormal chest X-ray, evidence of miliary TB.

H 224 records excluded because of missing data on HIV status.

I Immunosuppressed includes people immunocompromised because of a medical condition (e.g., leukaemia), or immunosuppressive therapy (e.g., prolonged use of high-doses of corticosteroids).

J Missed contacts are those with a known contact of another person with TB in the previous 2 years who was not evaluated or treated with latent TB infection or TB at that time.

K 1 record excluded because of missing data on site of disease.

L Limited to persons with TB disease 2010–2019 because purified protein derivative test date was not recorded before 2010 and IGRA test result or test date was not recorded before 2010. 12 records excluded because of missing data on treatment start.

M 22 records excluded because of missing data on initial treatment, including PZA.

N 149 records excluded because of missing data on outcome of therapy.

O Other reasons included refused, adverse events, moved, and unknown or unspecified reasons. Move was not considered an outcome of treatment after 2009.

P Limited to 2014–2019 because of data availability.

TB diagnostic findings differed by pregnancy status. Peri-pregnant people were more likely to have a positive IGRA and/or TST reported (79% vs. 73%, *P* < 0.001, Table 2) and 1.6 times more likely to have pulmonary TB disease than nonpregnant people (95% CI [1.4–1.8]); peri-pregnant people were more likely to have a CXR consistent with TB disease (87% vs. 77%, *P* < 0.001, Table 2). Additionally, more peri-pregnant people had a longer interval (90 days or more) between positive IGRA/TST and treatment start than peri-pregnant people (36% and 23%, respectively, *P* < 0.001). There was no difference in severity of TB disease between peri-pregnant and nonpregnant people based on presence of cavities on CXR, miliary, or meningeal TB, or adverse treatment outcomes. Death was more common among nonpregnant persons (2.4% vs. 0.3%, *P* < 0.001). Peri-pregnant people were more likely to be associated with an outbreak compared to nonpregnant people (*P* < 0.001), although only eight patients were identified as outbreak-associated (Table 2).

When classified by timing of pregnancy and TB treatment start (Table 3), we found that pregnant and postpartum people were more likely to have a reported positive IGRA/TST than pre-pregnant people (82% vs. 75%, *P* = 0.013). Pre-pregnant people were more likely to have positive sputum smears (48%) than pregnant and postpartum people (32%; *P* < 0.001). Similarly, pre-pregnant people were more likely to have culture-confirmed TB disease than pregnant and postpartum people (84% vs. 72%, *P* < 0.001). Pregnant and postpartum people were 2.3 times more likely to have a TB diagnosis that was not microbiologically confirmed than pre-pregnant people (95% CI [1.7–3.2]). However, pregnant and postpartum people were more likely to have a CXR consistent with TB disease (90% vs. 83%, *P* < 0.001, Table 3). Initial treatment including pyrazinamide was given to 94% of pre-pregnant and postpartum people compared to only 41% of pregnant people (*P* < 0.001). Use of pyrazinamide among pregnant people declined from 42% during 1993–2015 to 28% during 2016–2018, although the decline was not statistically significant (*P* = 0.2). Treatment completion was higher among pre-pregnant persons (96% vs. 92%, *P* = 0.003).

**Table 3.** Demographic and clinical characteristics by treatment timing.

Characteristic	Pre-pregnant, N = 451^A^	Pregnant and postpartum, N = 637^A^	*P* value^B^
Age			0.2
15–18	30 (6.7%)	36 (5.7%)	
19–25	162 (36%)	238 (37%)	
26–30	153 (34%)	177 (28%)	
31–35	73 (16%)	127 (20%)	
36–40	30 (6.7%)	53 (8.3%)	
41–45	3 (0.7%)	6 (0.9%)	
46–49	0 (0%)	0 (0%)	
Race and ethnicity^C^			0.057
American Indian/Native Alaskan	0 (0%)	1 (0.2%)	
Asian	169 (37%)	208 (33%)	
Black	42 (9.3%)	41 (6.4%)	
Hispanic	218 (48%)	362 (57%)	
Multiracial	0 (0%)	0 (0%)	
Native Hawaiian/Pacific Islander	5 (1.1%)	5 (0.8%)	
White	17 (3.8%)	20 (3.1%)	
Nativity^D^			0.003
Non-US born	352 (78%)	542 (85%)	
US born	99 (22%)	95 (15%)	
Time in the USA (non-US born)^E^			0.6
<2 years	111 (33%)	180 (35%)	
2–5 years	79 (23%)	128 (25%)	
Over 5 years	149 (44%)	211 (41%)	
Region			0.3
Bay Area	101 (22%)	172 (27%)	
Northern	2 (0.4%)	1 (0.2%)	
Central	56 (12%)	85 (13%)	
Border	49 (11%)	74 (12%)	
Southern	90 (20%)	126 (20%)	
Los Angeles	153 (34%)	179 (28%)	
TST and/or IGRA positive	340 (75%)	520 (82%)	0.013
Sputum smear positive^F^	185 (48%)	187 (32%)	<0.001
Microbiologically confirmed^G^	379 (84%)	459 (72%)	<0.001
Chest X-ray consistent with TB^H^	370 (83%)	568 (90%)	<0.001
Chest X-ray, evidence of a cavity^I^	88 (26%)	106 (21%)	0.09
Chest X-ray, evidence of miliary TB^J^	2 (3.1%)	1 (1.0%)	0.56
TNF antagonist treatment	0 (0%)	0 (0%)	>0.9
Post organ transplant	0 (0%)	1 (0.2%)	>0.9
Diabetes	8 (1.8%)	6 (0.9%)	0.2
End-stage renal disease	0 (0%)	0 (0%)	>0.9
Immunosuppressed^K^	1 (0.2%)	3 (0.5%)	0.6
Missed contact^L^	1 (0.2%)	0 (0%)	0.4
Site of disease^M^			0.090
Extra-pulmonary only	102 (23%)	110 (17%)	
Pulmonary and other	26 (5.8%)	39 (6.1%)	
Pulmonary only	323 (72%)	488 (77%)	
Meningeal TB	3 (0.7%)	1 (0.2%)	0.3
*Mycobacterium bovis*	5 (1.1%)	1 (0.2%)	0.087
≥90 days between positive IGRA/TST and treatment start^N^	42 (70%)	62 (60%)	0.2
Initial treatment includes PZA^O^	421 (93%)	392 (62%)	<0.001
Died prior to or during treatment	1 (0.2%)	2 (0.3%)	>0.9
Completed treatment^P^	433 (96%)	583 (92%)	0.003
Lost	7 (1.6%)	17 (2.7%)	0.2
Other reason for incomplete treatment^Q^	10 (2.2%)	35 (5.5%)	0.007
Outbreak-associated^R^	2 (0.4%)	5 (0.8%)	0.7

TST = tuberculin skin test; IGRA = interferon-gamma release assay; TNF = tumour necrosis factor; PZA = pyrazinamide.

A n (%).

B Fisher’s exact test for count data with simulated *P* value (based on 2,000 replicates); Pearson’s χ² test; Fisher’s exact test.

C 39 records excluded because of missing data on race and ethnicity.

D 33 records excluded because of missing data on nativity.

E Limited to those born outside the USA. 230 records excluded because of missing data on time in the USA.

F Limited to persons with pulmonary disease. 23 records excluded because of missing data on sputum smear status.

G 56 records excluded because of missing data on microbiologically confirmed.

H 212 records excluded because of missing data on chest X-ray abnormal, consistent with TB.

I Limited to persons with pulmonary disease and an abnormal chest X-ray consistent with TB disease. Five records excluded because of missing data on abnormal chest X-ray, evidence of a cavity.

J Limited to persons reported with an abnormal chest X-ray consistent with TB disease and whose TB disease was reported from 2010 to 2019 because data on abnormal chest X-ray, evidence of miliary TB, was not collected before 2010. 1 record excluded because of missing data on abnormal chest X-ray, evidence of miliary TB.

K Immunosuppressed includes people immunocompromised because of a medical condition (e.g., leukaemia), or immunosuppressive therapy (e.g., prolonged use of high-doses of corticosteroids).

L Missed contacts are those with a known contact of another person with TB in the previous 2 years who was not evaluated or treated with latent TB infection or TB at that time.

M 1 records excluded because of missing data on site of disease.

N Limited to persons with TB disease 2010–2019 because purified protein derivative test date was not recorded before 2010 and IGRA test result or test date was not recorded before 2010. 1 record excluded because of missing data on 90 days or more between positive IGRA/TST and treatment start.

O 22 records excluded because of missing data on initial treatment, including PZA.

P 149 records excluded because of missing data on outcome of therapy.

Q Other reasons included refused, adverse events, moved, and unknown or unspecified reasons. Move was not considered an outcome of treatment after 2009.

R Limited to 2014–2019 because of data availability.

## DISCUSSION

We identified 1,088 people in California who had TB during or soon after pregnancy. Overall, 7% of female people of childbearing age with TB during the period were pregnant. Seventy-three percent of the peri-pregnant cohort was under age 30; in contrast, 56% of Californian mothers were under 30 in the same period. We also found that 53% of the pre-pregnant cohort was Hispanic compared to 49% of California mothers.^14^ Peri-pregnant patients made up a higher proportion of TB patients particularly in central and border regions of California, where there are higher proportions of Hispanic people. Finally, peri-pregnant people were more likely to have arrived in the USA recently, which is consistent with findings from Demark and the UK.^11,15^

In our low-incidence setting, there was no difference in TB disease severity among peri-pregnant and nonpregnant patients; the higher death rate among nonpregnant is likely related to older age among this group. Peri-pregnant people were more likely to have TB diagnoses without a positive *Mycobacterium tuberculosis* culture and had 1.6 times higher rate of pulmonary TB, while nonpregnant people were twice as likely to have culture positive TB regardless of their site of disease. Peri-pregnant people were more likely to have a positive TST/IGRA reported 90 days or more prior to starting treatment. Our findings agree with previous reports that pregnancy does not increase disease severity if TB is diagnosed and treated in a timely manner.^12,15,16^ Data on the frequency of pulmonary versus extra-pulmonary TB among pregnant people are lacking; small European studies have reported higher rates among women and during pregnancy.^10,11,17^ Our higher rates of peri-pregnant people with pulmonary-only TB disease and fewer positive cultures might be a result of the proactive screening and evaluation of pregnant people for pulmonary TB in California. Pregnancy creates an added opportunity for access to medical care during which TB screening occurs. Previous publications have found active TB screening to identify TB at an earlier stage and with less severe disease,^18^ which might be reflected in our findings.

When comparing across pregnancy categories, we found that these differences in TB disease characteristics were driven by differences among people in the pregnant and postpartum groups, while the pre-pregnant group more closely resembled the nonpregnant group. Because pre-pregnant women are diagnosed with TB prior to finding out they are pregnant, their TB diagnosis and treatment is not influenced by pregnancy concerns. Therefore, our finding that pregnant and postpartum people were twice as likely to lack microbiological confirmation of TB disease as pre-pregnant people could suggest that TB screening among those already pregnant might have resulted in earlier TB diagnoses. Because TB is more difficult to diagnose during pregnancy,^3,7^ provider awareness and screening patients according to TB risk might lead to the earlier diagnosis or over-diagnosis of TB.

Treatment completion was over 90% in all groups. We did not find statically higher rates of pregnant women being lost to follow-up as was reported in Brazil where 19.1% of pregnant women were lost to follow-up compared to 12.2% of nonpregnant women.^19^ Fewer pregnant and postpartum patients completed treatment; although these numbers were small and we could not determine a specific cause based on changes in outcome variables over the period, this finding emphasises the importance of careful follow-up of pregnant women with TB to try to ensure continuation of therapy. In California, an average of 10% of patients with TB die each year; this cohort of women aged 15–49 is younger and likely healthier than our overall population of TB patients. Several studies in sub-Saharan Africa point to TB as a major cause of maternal mortality during pregnancy, especially among those co-infected with HIV^20,21^; however, our population had a low frequency of HIV infection.

We found that pyrazinamide use in California over this period aligned with the Centers for Disease Control guidelines that recommended providers not use this medication among pregnant patients.^22^ Although TB treatment guideline language softened in 2016,^23^ our data did not show a change in pyrazinamide use for pregnant patients after 2016. We found high pyrazinamide use for the initial treatment of TB among nonpregnant, pre-pregnant, and postpartum patients (94%, 95%, and 95%, respectively); conversely, only 40% of pregnant people started on TB treatment that included pyrazinamide.

Finally, we identified TB prevention opportunities among pregnant people. Overall, 24% of nonpregnant people and 36% of peri-pregnant people had a positive TST/IGRA reported more than 90 days prior to their TB treatment start date, which might suggest that they had diagnosed LTBI that subsequently progressed to active TB. People in the postpartum group had the highest proportion of reported positive TST/IGRA 90 days or more prior to TB treatment start, which might further suggest that treatment for LTBI was deferred until after pregnancy; TB symptoms for these people might have developed before LTBI treatment could be started in the recommended 2–3 months following delivery.^13^ Alternatively, the positive TST/IGRA could have been obtained months to years prior to pregnancy, in which case the failure to treat previously diagnosed LTBI could represent a missed opportunity to prevent TB progression during and following pregnancy.

Our study had limitations. We used a birth record match to identify people with live births, but this might have miscategorised people with pregnancy losses as nonpregnant. We assumed that a TB treatment start date more than 90 days after a positive TST/IGRA would be unrelated to evaluation resulting from that positive test; we are likely underestimating this population as positive TSTs or IGRAs were not reportable during the study period. Finally, some surveillance variables were added during the period, allowing for inclusion of the variables only during later years of the cohort, while other variables (such as HIV) were much more commonly reported earlier in the period, limiting comparisons across the 30-year period. Notably, TB surveillance data in the USA yield highly representative population-based data.^24^

This is the first attempt to describe the frequency and characteristics of pregnant patients with TB in California. A high proportion of young people with TB and pregnancy identify as Hispanic, which suggests a need for coordination between TB care programmes and obstetric providers who serve Hispanic people, especially recent arrivers. Care coordination can ensure adequate screening as well as optimal and timely treatment for TB disease and LTBI treatment. We found that many people had more than 3 months between initial positive TST/IGRA and TB disease treatment, which could point to an opportunity for some pregnant people to receive LTBI treatment and avoid progression to active TB disease. New national guidelines are more supportive of treating LTBI during pregnancy using rifampin, which could prevent postpartum TB disease.^25^ We can use these data to reinforce the importance of screening for TB disease before or during pregnancy. LHDs, especially those with higher proportions of pregnant TB patients, can engage local obstetrical providers to encourage TB screening. These data provide an important baseline for newer TB surveillance that captures pregnancy at the time of TB diagnosis.
